# Exploring reasons for COVID-19 vaccine hesitancy among healthcare providers in Ethiopia

**DOI:** 10.11604/pamj.2021.40.213.30699

**Published:** 2021-12-08

**Authors:** Fisseha Shiferie, Oumer Sada, Theodros Fenta, Mirgissa Kaba, Atalay Mulu Fentie

**Affiliations:** 1 Department of Epidemiology and Biostatistics, Addis Continental Institute of Public Health, Addis Ababa, Ethiopia,; 2 Department of Pharmacology and Clinical Pharmacy, Addis Ababa University, College of Health Sciences, School of Pharmacy, Addis Ababa, Ethiopia,; 3 Department of Medicine Registration and Licensing Directorate, Ethiopian Food and Drug Authority, Addis Ababa, Ethiopia,; 4 Department of Preventive Medicine, School of Public Health, Addis Ababa University, Addis Ababa, Ethiopia

**Keywords:** COVID-19 vaccine, healthcare providers, vaccine hesitancy, Ethiopia

## Abstract

**Introduction:**

the World Health Organization has identified vaccine hesitancy as one of the top ten threats to global health. The purpose of this study was to explore factors contributing to COVID-19 vaccine hesitancy among healthcare providers, their perspectives regarding vaccine uptake by the public and their recommendations to improve vaccine uptake in Ethiopia.

**Methods:**

a phenomenological qualitative study was conducted among purposively selected healthcare providers working in the Ministry of Health (MoH), regulatory authority, public and private hospitals and health centres who hesitated to take the COVID-19 vaccine in Addis Ababa, Ethiopia in June 2021. A total of twenty in-depth interviews were conducted using a semi-structured open-ended interview guide. Participants included nurses, physicians, pharmacists, health officers, Medical Laboratory technologists and midwives. A qualitative content analysis approach was chosen to analyse the data.

**Results:**

all the participants agreed (n=20) that lack of consistent information and inadequate evidence about COVID-19 vaccine safety, efficacy and quality were the main reasons for COVID-19 vaccine hesitancy. History of perceived and confirmed COVID-19 infection history, misinformation, religious views, unknown short and long-term effects of the vaccine and undefined length of time of vaccine´s protection were also other reasons mentioned by the participants.

**Conclusion:**

healthcare providers were hesitant toward COVID-19 vaccine mainly due to lack of clear evidence regarding the vaccine´s short and long-term safety, efficacy and quality profiles. Hence, the long-term safety and efficacy of the vaccine should be extensively studied and evidence dissemination and communication should be clear and transparent.

## Introduction

The COVID-19 disease is an extremely contagious respiratory illness caused by the novel coronavirus (SARS-CoV-2) and become a worldwide public health, economic and psychosocial threat [1]. As of August 31, 2021, more than 219 million COVID-19 cases and 4.54 million deaths were reported worldwide and 5.6 million cases and 136 thousand deaths in Africa. Similarly, in Ethiopia, about 310 thousand cases and 4692 deaths were reported [2]. Similar to the worldwide situation, the pandemic highly affected the Ethiopian economy and in the best-case scenario, the pandemic is expected to reduce the Ethiopian economy by 6.5% whereas in the worst-case scenario, the pandemic may reduce the GDP by 16.7% [3]. Efforts have been made to prevent and control its spread around the globe. Since there are no specific treatment options available, preventive strategies recommended by World Health Organization (WHO) are proven to be effective [4]. Alongside with public health measures in place, the speed for the invention of a vaccine against COVID-19 has been much quicker than any other vaccines invented in human history because of its urgent need to control the pandemic [5].

According to the World Health Organization (WHO) report, there are currently more than sixty COVID-19 vaccine candidates in clinical development and over 170 in pre-clinical development [6]. Some successful vaccines against COVID-19 have already been publicized and were approved for emergency use within a year [7,8]. AstraZeneca's COVID-19 vaccine is one of the approved vaccines for emergency use by WHO´s Emergency Use Listing and are being distributed and administered across several countries including Ethiopia [7]. Based on the vulnerability and risk of contracting the virus, Ethiopia provided 2.2 million doses of the vaccine obtained by donation for healthcare workers freely [9]. Despite its distribution and administration in many countries, hesitation to take the vaccine is becoming a major challenge [10]. Vaccine hesitancy is not a problem of some specific countries, rather, it is a worldwide problem. The WHO has identified vaccine hesitancy as one of the top ten threats to global health in 2019 [11]. The (SAGE) working group defined vaccine hesitancy as “delay in acceptance or refusal of vaccination despite availability of vaccination services.” Vaccine hesitancy is complex and context specific, varying across time, place and vaccine type and influenced by factors such as complacency, convenience and confidence [12].

Healthcare providers are key individuals in building vaccine confidence and increasing acceptance of the public by providing information about safety and efficacy of the vaccines, convincing and motivating others by sharing their experiences and personal reasons to be vaccinated [13]. COVID-19 vaccine hesitancy among healthcare providers has not been well studied in low- and middle-income countries (LMICs) like Ethiopia, indicating the need to explore reasons for vaccine hesitancy. Hence, the purpose of this phenomenological qualitative study was to explore factors contributing to COVID-19 vaccine hesitancy among healthcare providers, their perspectives regarding vaccine uptake by the public and their recommendations to improve vaccine uptake in Ethiopia.

## Methods

**Study design and period:** a phenomenological qualitative study designed was employed, using an in-depth interview to understand the perceptions and experiences of healthcare providers towards COVID-19 vaccine hesitancy. Interviews were conducted from June 6 through 19, 2021.

**Study area:** the study was conducted in the capital city of Ethiopia, Addis Ababa. According to the Central Statistics Agency´s population projection, the population of Addis Ababa is more than 5.0 million with increment of 4.42% from 2020 [14]. Addis Ababa has a total of 38 hospitals (13 public and 25 private hospital), 97 health centres, 343 speciality clinics, 458 medium clinics and 179 primary clinics. There are about 715 physicians, 868 pharmacy professionals, 4355 nurses, 1093 health officers and 797 medical laboratory technologists.

**Data collection:** a face-to-face and virtual in-depth interview was conducted among purposively selected healthcare professionals (Physicians, Nurses, Health officers, Midwives, Pharmacists and Medical laboratory technologists) working in public and private hospitals, clinics, academia, regulatory authority and Ministry of Health who hesitated to take the first dose of COVID-19 vaccine. The different sectors mentioned earlier whose healthcare providers had access to the vaccine were first identified, and then every healthcare provider was approached and asked whether they were vaccinated or not. If they hesitated and were not vaccinated, their oral consent was obtained before they were interviewed. Efforts were made to keep the variety by including health care professionals from different departments and sectors.

A refined and pre-tested interview guide was used to interview participants. Participants involved in the pretest were those health care providers who hesitated to take the COVID-19 vaccine, and they were not included in the final analysis. Besides, after the pretest, the interview guide was modified where necessary. Probing questions were included in case the responses of the participants were superficial and/ the answers were conflicting.

Interview questions were classified into three themes. The first theme focused on exploring their view and reasons for COVID-19 vaccine hesitancy. The second theme was designed to understand healthcare providers´ perspectives regarding vaccine uptake by the public. The third theme was aimed at exploring healthcare providers´ recommendation to improve the acceptance of COVID-19 vaccine by healthcare providers themselves and the public at large. Four Ph.D. students who got a special training on qualitative research conducted the interview. The average duration of the interview was 20 minutes, ranging from 10 to 25 minutes. Data collection was stopped when data saturation was perceived to be reached. Interviews were tape recorded and notes were also taken. The interview was done in Amharic and any ambiguities raised from the interviewees were cleared at the time of the interview.

**Data management and analysis:** thematic content analysis approach was chosen to analyse the data. All audio-recorded Amharic versions of the interviews were transcribed verbatim by the respective data collectors and translated back to English. Experienced translators who had years of experience in qualitative research did the translation, four independent reviews were made and later validated by the senior author. The transcripts were intensively read text by text to well understand the data, the data was manually coded and themes emerged. Furthermore, findings of the study were communicated to some study participants for authenticity of interpretations.

**Ethical consideration:** ethical clearance was obtained from Department of Preventive Medicine, School of Public Health, College of Health Sciences, Addis Ababa University (Ref. Prev/68/2021) and verbal consent was secured from each participant prior to data collection. In reporting the findings, codes were used to maintain anonymity of participants and confidentiality of the information.

## Results

**Figure 1:**
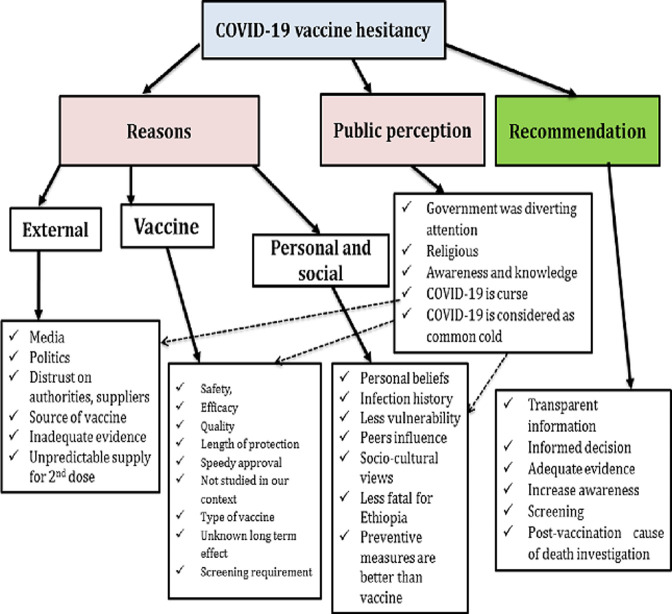
the major themes emerged from the in-depth interviews

A total of twenty in-depth interviews were conducted. Five nurses, four physicians, four pharmacists, three health officers, two medical laboratory technologists and two midwives participated in the study. Half of them were females and ages ranged from 27 to 45 years. Three major themes emerged from the in-depth interviews: reasons for COVID-19 vaccine hesitancy, COVID-9 vaccine uptake from the perspective of healthcare providers and their recommendations to improve COVID-19 vaccine acceptance. There are emerging sub-themes within each major theme (Figure 1).

**Reasons for COVID-19 vaccine hesitancy:** the three sub-themes which emerged out of the reasons for COVID-19 vaccine hesitancy include COVID-19 vaccine related factors, external factors and personal & social factors.

**COVID-19vaccine related factors:** all the participants agreed (n=20) that inadequate evidence about the disease itself and the vaccine´s safety, efficacy and quality in association with its speedy approval were the major reasons for not taking the COVID-19 vaccine. The presence of various types of COVID-19 vaccine, unknown long and short-term effects and undefined length of time of the vaccine´s protection also contributed their parts in developing vaccine hesitancy by the participants.

***COVID-19 vaccine safety:*** reports such as cerebral venous sinus thrombosis, splanchnic vein and arterial thrombosis, together with low levels of blood platelets and sometimes bleeding due to the COVID-19 vaccine were the major reasons for vaccine hesitancy. One participant stated that (VH001):

*“As soon as the COVID-19 vaccine arrived in Ethiopia, the risk of blood clot was the news here and there especially by the social media. Due to these reasons, I refused to be vaccinated against COVID-19”*.

Another female study participant also added that (VH0015):

*“The main reason why I refused to be vaccinated is because of fear of side effects especially due to the cerebral thrombosis”*.

Apart from the vaccine hesitancy associated with adverse effects reported by different printed and electronic media abroad and locally, some of the participants were also mentioned the actual experiences they encountered.

*“One of my colleagues died four days after he received the COVID-19 vaccine which left all of us shocked. We concluded that the cause of his death was related to COVID-19 vaccine and as a result, most of my colleagues including myself decided not to take the vaccine (VH008)”*.

VH006 also added that:

*“My sister who is a healthcare professional received the vaccine before the vaccine was available in the hospital where I am currently working. I saw her suffering a lot for few days due to the side effects and I decided not to be vaccinated”*.

The very short time it took for the vaccine to be developed was also mentioned as one of the reasons that made them not to trust the vaccine. One of the participants who had a comorbid illness said that the long-term safety and complications were not studied well (VH004).

*“As per my readings and clinical experience, COVID-19 vaccine has autoimmune complications. The vaccine is immunogenic and may predispose and exacerbate my risk of developing immunologic diseases. As a result, I would rather prefer not to take the vaccine”*.

Participant VH009 also added that:

*“Based on the information I have most of the vaccines are nucleic acid based. This has made its long-term effects to remain unknown and I fear this might affect my own DNA replication”*.

***COVID-19 vaccine efficacy:*** more than half of the participants (n=11) were concerned about the vaccine´s efficacy. They unequivocally mentioned that all the approved vaccines were not well studied and there is a huge lack of evidence about the vaccine´s protection against the COVID-19 infection. They also stated that much remains unknown about the disease itself and the emerging variants. Moreover, they said that different countries reported a significantly different vaccine protection level for the same COVID-19 vaccine. For example, one of the study participants stated (VH0016) that:

*“The vaccine is not well studied and they are doing a clinical trial on us. We heard that different variants of COVID-19 are recently reported. This means if there are 50 different variants, I have to take 50 different vaccines. This is a clear sign that the disease is not well studied”*.

Another participant also supplemented that (VH0011):

*“They told us to continue the non-pharmacological prevention measures after taking the vaccine. That means, even if we take the vaccine, we will remain at risk of infection and we can also pass the virus to others. Therefore, this tells us that the people who produced the vaccine are not even sure about the effectiveness of the vaccine”*.

One participant also stated that the vaccine was not studied in our context.

*“I am not interested to take drugs unless they are studied in our context because genetics and other factors may contribute for the effectiveness and safety of the vaccine”*.

**Personal and social factors:** personal and social factors were also the other reasons mentioned by the participants for COVID-19 vaccine hesitancy.

***Personal beliefs:*** some of the participants perceived that COVID-19 had low fatality rate in Ethiopia compared to other countries. They also stated that preventive measures against COVID-19 infection are better than the vaccine. One participant articulated her perception towards the vaccine as follows (VH003):

*“The prevalence and the fatality rate of the disease in Ethiopia are not like western countries and the vaccine doesn´t provide 100 percent protection. Hence, I would rather keep using protective measures instead of being vaccinated. So, why I expose myself to vaccine related risks”*.

A healthcare professional who has been working at COVID-19 treatment center added (VH002):

*“Although I have been working at a COVID-19 treatment center for one month, I preferred the already existing preventive measures such as wearing face mask, social distancing, hand sanitizer etc. than taking the vaccine”*.

***Lack of trust on the vaccine:*** a significant number of participants (n=7) also were very suspicious of the speedy approval of the vaccine. They believed that both the disease and the vaccine are politicized. They also pointed out that the huge competition that exists amongst scientists, companies and countries towards COVID-19 vaccine development made them to remain very skeptical. One participant mentioned that (VH0012):

*“Chronic diseases such as HIV/AIDS from which the world is suffering from for a long period of time did not have a vaccine yet. However, it is very confusing for me that COVID-19 vaccine was developed in such a very short time”*.

One participant added that (VH003):

*“Countries around the world are producing their own vaccines. They also urge their people to take their own vaccine only. However, because we are poor and can´t produce our own vaccine, we are getting it from abroad. As a result, I am forced to believe that the vaccine producing countries are making money out of vaccines”*.

Moreover, lack of trust on the quality of donated and imported medicines and vaccines to LMICs like Ethiopia were frequently mentioned as reasons for vaccine hesitancy. Participants were very skeptical about the quality of COVID-19 vaccine and this hindered them from being vaccinated. One participant said (VH001):

*“Most donated medical products for developing countries have low quality and short shelf life. The products are imported with push system without checking their quality and safety. If a certain product is donated, the regulatory authority will simply make a skimmed inspection. They don´t strictly check their qualities as they do for other imported medical products”*.

***COVID-19 infection or contact history:*** participants believed that they have already developed immunity against the virus as they were already infected before the vaccine was introduced. VH0019 participant stated that:

*“I was already infected and recovered from the disease. So why should I take the vaccine? I believe that I have already developed immunity against the virus”*.

Another participant also stated that (VH0011):

*"I had all the symptoms for more than a week even if I was not tested. I believe that I was infected and recovered. If this is the case, why should I take the COVID-19 vaccine?”*.

***Less vulnerability:*** participants also tried to associate their ages and current health status with the risk of contracting the virus. One participant stated as:

*I believe that I am less vulnerable to the virus as I am young and do not have any other comorbidity*.

### External factors

***Misinformation:*** information that is being disseminated via various media outlets about COVID-19 vaccine has also affected the acceptance rate of the vaccine by the participants. One participant mentioned that (VH001):

*“The reason why I didn´t receive the COVID-19 vaccine was because of the information I heard from a live discussion on an international media comparing the three different types of COVID-19 vaccines: AstraZeneca, Pfizer and Johnson and Johnson vaccines. During their discussion they said that AstraZeneca vaccine is not recommended for people under 35 years of age”*.

Some of the participants were also mentioned that information disseminated from exemplar or respected individuals made them hesitant to the vaccine. One study participant stated that (VH009):

*“As an academician, I spent too much of my time with senior Professors and realized that they did not want to be vaccinated against the virus. I thought that their decision was correct and I also decided not take the vaccine”*.

***Distrust on government authorities:*** before launching the vaccination campaign, the community needs to be well informed about the vaccine. The confusion and rumors around the vaccine that came after it was introduced should have been cleared as well. One participant stated (VH004):

*“Concerned government bodies like the Ministry of Health (MoH) and the Ethiopian Food and Drug Authority (EFDA) could have clearly communicated everything about the vaccine before/after it was introduced. Failing to do has left many individuals like me confused. How can I get vaccinated without having adequate knowledge about the vaccine?”*.

Unpredictable supply for the second vaccine dose has made many participants hesitant to be vaccinated. One of the participants said that (VH0016):

*“We are told that the vaccine has two doses. But I very much doubt about getting the second dose as the vaccine was obtained by donation. It is now 3 months since people got the first dose. I don´t know when they will get the second dose”*.

**Public perception towards COVID-19 vaccine:** all the participants uniformly stated that the community´s strong religious beliefs have affected the COVID-19 vaccine uptake. One of the participants stated that (VH004):

*“My family and neighbors told me not to take COVID-19 vaccine, because they associated it with microchips, illuminati (666) etc”*.

Participant VH0010 also stated that:

*“The vast majority of the community especially the elderly believes that COVID-19 disease is a curse from God for violating his rules. Hence, the only way to get out of this mess is through prayers”*.

People also underestimated the disease and they called it a common cold and tried to associate it as means of diverting political pressure. One participant said that:

*“Some people assumed that COVID-19 is like a common cold and it will go by itself and no need to take any vaccine. The government is simply trying to divert our attention away from the complex political situation our country is in”*.

**Healthcare providers´ recommendations to improve vaccine uptake in Ethiopia:** all the participants recommended both the disease and the vaccine (especially its long-term safety and efficacy) to be extensively studied. The manufacturers, WHO, government and other concerned bodies should be transparent in disseminating the correct information and evidences about the vaccine. They also recommended that responsible government bodies should go down to the healthcare facilities and the community in order to assess their demand before they tell people and the healthcare providers to get vaccinated. In other words, awareness creation should be done beforehand. Even after the introduction of the vaccine, questions that are being raised by the community regarding the vaccine should be handled and addressed properly.

## Discussion

Vaccine hesitancy is affecting the global attempt towards controlling the current COVID-19 pandemic. Understanding the perceptions of healthcare professionals about the COVID-19 vaccine and working to improve the acceptance rate would help to limit the consequences of the pandemic [15,16]. In low- and middle-income countries, vaccination is generally accepted, with pockets of reduced acceptance [17]. Vaccine hesitancy is prevalent in rich and poor regions and can be found among individuals of various socioeconomic, cultural, ethnic and religious backgrounds. Hesitancy can stem from marginalization and social exclusion, negative experiences at health services, misinformation about vaccines circulated on unregulated social media platforms or lack of trust in authorities and institutions [18].

The short- and long-term safety concerns were unequivocally mentioned as the major reasons for COVID-19 vaccine hesitancy by all participants of this study. Acknowledging any concerns associated with vaccines or any medicine related unwanted effects should be an important first step in any conversation with healthcare providers and the public. According to a study done by Jessica *et al*. explaining that all events that occur following vaccination may not be caused by the vaccine and providing easy access to medical advice when adverse effects occurred had a paramount importance in building trust and addressing concerns before they trigger a level of fear that amplifies the negative experience [19].

Participants also pointed out that misinformation about COVID-19 and the vaccine has affected the acceptance rate of the vaccine. Different studies showed that such misinformation can range from myths and unfounded speculations to targeted falsehood information rooted in conspiracy theories. People who are ill-informed about vaccines in the media will end up with reporting more adverse events, thereby increasing and validating other people´s concerns. Moreover, it is known that if misinformation left unchallenged, it can affect people´s perceptions and decision-making and subsequently can lead to a self-perpetuating cycle of negative news [17-20]. Any COVID-19 vaccine related misconceptions need to be properly addressed through public discourse before elevating them. However, providing correct information for those with higher levels of anxiety may not necessarily decrease vaccine hesitancy and other solutions need to be designed [21]. In this regard, the role of public health officials, religious leaders and especially healthcare workers in disseminating strong, clear, consistent messages that are accurate and align with standardized vaccination guidelines to combat misinformation/disinformation is encouraging but not enough.

Inadequate evidences associated with speedy approval of the COVID-19 vaccine, perceived fewer vulnerabilities to COVID-19 infection, undefined length of time of the vaccine´s protection, re-infection risks and peer influences were some reasons mentioned by participants for vaccine hesitancy. Expectations about vaccines and their effect on the COVID-19 virus were the filters of our perceptions and what determines these expectations is far broader than our knowledge. Decision-making about whether to take the vaccine are significantly influenced by peers, available evidences and our willingness to accept individual risks for the greater good [17-22].

In this qualitative study, public perceptions towards COVID-19 vaccine were explored through health care professionals. It clearly showed that the public had different perspectives on vaccination hesitancy in Ethiopia. Most of the participants mentioned that the public didn´t have clear information about the disease and the vaccine. In line with a previous study [23], the public perceived the disease as a curse from GOD because of our sins and the only solution they proposed to get rid of it is through prayers and repentance. As a result, they resisted taking any kind of vaccine. On the other hand, some of them associated it with the so-called Illuminati (666) religious group and others said that it is a microchip intended to control our day-to-day activities. Ethiopia is one of the few conservatively religious countries in the world. Spiritual explanations of health issues with regard to attribution, intervention and coping strategies tend to be more widely accepted than the biomedical model. Therefore, the religious and socio-cultural dimension of the population shouldn´t be ignored and need to be properly addressed [24].

There are few limitations in the present study. First, the data was collected only in Addis Ababa and might not reflect the perceptions of healthcare professionals who work out of the capital city of Ethiopia, Addis Ababa. The other limitation is that participants´ hesitancy to be vaccinated might change over time as subsequent shipments of vaccines arrive and more people get vaccinated and this might affect the different perceptions they had initially. Currently, the number of cases is also increasing at an alarming rate which might also change the perceptions of participants towards the vaccine. Due to the qualitative nature of the research, we can´t draw a statistical inference.

## Conclusion

It is a usual misconception that healthcare providers have a positive attitude towards vaccines because they have scientific and medical training. However, this is not always true and our study has explored the various reasons why healthcare providers like other members of the community were hesitant to be vaccinated. Therefore, it is important to further explore and understand reasons for COVID-19 vaccine hesitancy among healthcare providers to propose a meaningful solution to improve vaccine uptake. Healthcare professionals need to acknowledge all the suspected adverse effects and provide accurate and credible information about the vaccine´s safety and efficacy, proactively release timely information and data regarding vaccination strategies, modalities and accomplishments in disaggregated, user-friendly and open-source formats, enhance transparent and coherent public communication to address misinformation and the ‘infodemic’ and engage the public when developing vaccination strategies.

### What is known about this topic

- It is already known that vaccine hesitancy is a global public health problem;
- Like other members of the community, healthcare providers are also hesitant to get COVID-19 vaccine.

### What this study adds

- Our study has explored various reasons why healthcare providers in Ethiopia were hesitant to get the COVID-19 vaccine;
- Some of these reasons have never been explored by previous studies done so far;
- We have also tried to explore the perceptions of the public towards COVID-19 vaccine through healthcare providers.
